# True Ileal Amino Acid Digestibility and Protein Quality of ^15^N-Labeled Faba Bean in Healthy Humans

**DOI:** 10.1016/j.tjnut.2024.01.030

**Published:** 2024-02-03

**Authors:** Suvi T Itkonen, Juliane Calvez, Gheorghe Airinei, Martin Chapelais, Nadezda Khodorova, Moulay Sahaka, Robert Benamouzig, Frederick L Stoddard, Asko Simojoki, Anne-Maria Pajari, Claire Gaudichon

**Affiliations:** 1 Université Paris-Saclay, AgroParisTech, INRAE, UMR PNCA, Palaiseau, France; 2 Department of Food and Nutrition, University of Helsinki, Helsinki, Finland; 3 Department of Agricultural Sciences, University of Helsinki, Helsinki, Finland

**Keywords:** ^15^N faba beans, healthy volunteers, intestinal tubes, true ileal digestibility, DIAAS, nitrogen retention

## Abstract

**Background:**

The recommended transition toward more plant-based diets, particularly containing legumes, requires a wider knowledge of plant protein bioavailability. Faba beans are cultivated at different latitudes and are used increasingly in human nutrition.

**Objectives:**

We aimed to assess the nutritional quality of faba bean protein in healthy volunteers equipped with an intestinal tube to implement the ileal ^15^N balance method.

**Methods:**

Nine volunteers completed the study (7 males, 2 females, aged 33 ± 10 y, BMI: 24.7 ± 2.6 kg/m^2^). They were equipped with a nasoileal tube. After fasting overnight, they ingested a test meal consisting of cooked mash of dehulled faba bean seeds (20 g protein per serving of approximately 250 g) intrinsically labeled with ^15^N. Samples of ileal contents, plasma, and urine were collected over an 8-h postprandial period. Undigested nitrogen (N) and amino acids (AAs) were determined using isotopic MS, and subsequently, ileal digestibility and digestible indispensable amino acid score (DIAAS) were calculated. The measurement of postprandial deamination allowed calculation of the net postprandial protein utilization (NPPU).

**Results:**

The ileal N digestibility was 84.1% ± 7.7%. Postprandial deamination represented 19.2% ± 3.6% of ingested N, and the NPPU was 64.7% ± 9.7%. The ileal digestibility of individual AAs varied from 85.1% ± 13.7% for histidine to 94.2% ± 3.6% for glutamine + glutamate. The mean AA digestibility was ∼6 percentage points higher than the digestibility of N, reaching 89.8% ± 5.9%, whereas indispensable AA digestibility was 88.0% ± 7.3%. Histidine and tryptophan were the first limiting AAs [DIAAS = 0.77 (calculated by legume-specific N-to-protein conversion factor 5.4); 0.67 (by default factor 6.25)]. Sulfur AAs were limiting to a lesser extent [DIAA ratio = 0.94 (N × 5.4); 0.81 (N × 6.25)].

**Conclusions:**

Protein ileal digestibility of cooked, dehulled faba beans in humans was moderate (<85%), but that of AAs was close to 90%. Overall protein quality was restricted by the limited histidine and tryptophan content.

This trial was registered at clinicaltrials.gov as NCT05047757.

## Introduction

Decreasing the consumption of animal-based protein and increasing the consumption of plant-based protein will support the transition toward more sustainable food systems and healthier diets [1]. Plant-based diets have been associated with lower risk of chronic diseases, such as cardiovascular disease, obesity, type 2 diabetes, and certain cancers, which may be attributable to higher intakes of nutritionally beneficial substances, for example, fiber [2,3]. However, a transition toward more plant-based diets may alter protein security, and thus, it requires a good knowledge of plant protein and amino acid (AA) bioavailability. Indeed, there is concern about whether increasing the proportion of plant protein in the diet will lead to inadequate intake of protein and indispensable amino acids (IAA), in addition to the question about protein bioavailability [4]. Generally, digestibility of plant-based proteins in their original food matrix is considered lower (70%–85%) than that of animal-based sources (90%–95%) [5].

The FAO of the United Nations [6] recommends the use of the digestible indispensable amino acid score (DIAAS) to evaluate protein quality. DIAAS takes into account the ileal digestibility of the individual AAs, whereas the protein digestibility corrected amino acid score (PDCAAS) tracks only the total nitrogen digestibility. The standard method to measure ileal digestibility is the ileal balance method, which requires the collection of ileal digesta using an intestinal tube. Together with the intrinsic labeling of dietary protein with a stable isotope, it allows determining the ileal digestibility of nitrogen and AAs, with strengths and limitations that have been described in detail by Bandyopadhyay et al. [7]. As this method is invasive, an alternative, minimally invasive method, that is, the dual isotope method, has been developed to determine AA digestibility [8,9]. It has provided AA digestibility values for several legumes. For whole chickpeas and yellow peas, digestibility values were 72%–75%, and dehulling improved AA digestibility of mung bean from 63% to 71% [9]. Until now, studies on legume digestibility in humans using the ileal balance method have mainly focused on flours, isolates, and concentrates [[10], [11], [12], [13]], so there is room for investigating the digestibility of cooked, dehulled beans. The intrinsic labeling of plants with ^15^N allows not only to determine the digestive losses of nitrogen but also the metabolic losses through deamination and, in turn, the postprandial nitrogen retention, so-called net postprandial nitrogen utilization (NPPU) [[14], [15], [16], [17]].

Faba bean (*Vicia faba* L., also known as broad bean or horse bean) is cultivated on all inhabited continents and is adapted to cool climates where, for example, soybeans do poorly [18], making faba bean a potential source of protein for wider use. Faba bean AA profile is relatively well balanced depending on the cultivar; usually, the concentrations of sulfur AAs, such as methionine and cysteine, as well as tryptophan, are low [19]. Nowadays, faba bean is used as an ingredient in many processed plant-based foods, such as meat alternatives, which are becoming increasingly popular [[20], [21], [22]]. Thus, it is timely to investigate faba bean protein quality in more depth.

In the current study, we aimed to assess dehulled and cooked faba bean true ileal AA and nitrogen digestibility, NPPU, and DIAAS in healthy volunteers using an ileal ^15^N balance method.

## Materials

### Study design

This study was conducted as a Finnish-French collaboration in the multidisciplinary project Leg4Life (Legumes for Sustainable Food System and Healthy Life), funded by the Strategic Research Council at the Research Council of Finland. The study was approved by the Ethics Committee Ile de France VI on July 12, 2021 (ref. 2020-A02888-31) and was registered at clinicaltrials.gov as NCT05047757. The clinical trial was conducted in the Human Nutrition Research Center of Avicenne Hospital (APHP) (Bobigny, France) in accordance with the ethical standards of the responsible committee on human experimentation. The subjects consumed dehulled and cooked ^15^N-labeled faba beans served as a mash. Nitrogen and AA true ileal digestibility and NPPU of faba bean protein were the primary outcomes.

### Faba bean cultivation and ^15^N labeling

Faba beans were labeled intrinsically by fertilization with ^15^N in the field. The soil surface area of a 20-m^2^ plot of faba beans (*Vicia faba* cv. Sampo) was fertilized with 3 doses of ^15^N-labeled ammonium nitrate (^15^NH_4_^15^NO_3_; 10%) solution containing 3.5 g of ^15^N per plot at 2-wk intervals between flowering and pod filling in early July to early August 2020. The labeling protocol was carried out at Viikki Research Farm by the Department of Agricultural Sciences (University of Helsinki, Finland). The whole biomass of faba bean was harvested at maturity in late mid-September 2020, pooled, and dried in a cold-air drier for 2 d, after which the seeds were further dried at 30°C–40°C in a force-ventilated oven for 1 d. Nitrogen content of dry bean was 5.04% (protein content 27.2 g/100 g, N × 5.4), and ^15^N enrichment was 2344 δ‰, that is, 1.21 atom percent (AP).

### Meal preparation

Dehulled beans (75 g per 250 g meal, fresh matter) were soaked overnight and rinsed. They were cooked for 30 min, and vegetable bouillon powder was added to provide a salt content of 0.7% in the final product. The faba beans were then mashed with a blender to yield a smooth texture, and the mash was frozen. The preparations were carried out at the Department of Food and Nutrition, University of Helsinki, Finland.

The N% and ^15^N enrichment were measured in the beans before and during the preparation in 5 replicates, as shown in Supplemental Table 1. The calculated amount of bean mash providing 20 g protein (3.7 g nitrogen, N × 5.4) was 234.1 g.

### Subjects

All subjects were required to be in good general health. Inclusion criteria were as follows: adult of either sex, BMI of 18–30 kg/m^2^, and age of 18–65 y. Exclusion criteria were any food or latex allergy, positive serology for HIV, hepatitis B and C viruses, and COVID-19, blood donation within the last 3 mo, anemia, pregnancy, abusive drug or alcohol consumption, severe disease, or sports activity of >7 h/wk. All subjects received detailed information on the purpose and potential risks of the study protocol. A written informed consent was obtained from all subjects.

### Experimental protocol

Each volunteer received a 250 g bean mash meal. Before serving, the meal was thawed and heated in a microwave, and 10 g of unsalted butter was added. The subjects were not allowed to ingest any other food during the 8-h postprandial sampling period, except for the hourly mandatory glass of water. After 8-h, the residual amount of undigested meal is considered negligible (corresponding to <0.5% of the ingested N), as observed in our previous studies [13,[15], [16], [17]].

**FIGURE 1:**
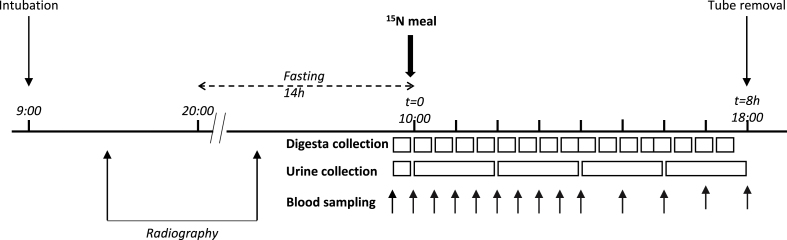
Experimental protocol.

Figure 1 shows the experimental protocol. Six days before the experiment, the volunteers were instructed to follow dietary advice to standardize protein intake at ∼1.2 g protein/(kg per day), corresponding to the average intake of the French population [23]. The subjects were hospitalized for 2 d. On the first morning, a triple-lumen intestinal tube was administered under local anesthesia through the nose by a trained gastroenterologist. The tube was allowed to migrate along the digestive tract for 24 h. The last meal was served at 20:00, before the overnight fast (14 h). On the morning of the second day, the position of the tube was checked by radiography. The correct position in the terminal ileum was also ensured by measuring the pH of the intestinal fluid (digesta), which is around 8. Polyethylene glycol 4000 (PEG-4000; 20 g/L) was used as a nonabsorbable marker to measure the intestinal flow rate. It was perfused with a flow rate of 1 mL/min, 20 cm above the digesta collection zone. Digesta was collected for 30 min before the meal, and the collection was continued for the 8-h postprandial period. The sample was pooled every 30 min, and a protease inhibitor (diisopropyl fluorophosphate) was added. Thereafter, the sample was frozen at −20°C and lyophilized until analysis. For blood sampling, a catheter was placed into the forearm vein. A baseline blood sample was drawn before the meal and the sampling was performed every 30 min for the first 4 h and hourly thereafter. Urine was collected every 2 h, weighed, and stored at −20°C. For urea extraction, a fraction of urine was stored at 4°C with thymol oil and liquid Vaseline and processed on the next day.

Eighteen participants were recruited from October 20, 2021, to January 12, 2023, among whom only 9 completed the study. The intestinal tube was removed before the end of the experimental protocol for different reasons in 9 participants: *1*) painful (*n* = 2), *2*) vomiting (*n* = 1), *3*) nonmigration through the pylorus (*n* = 5), and *4*) technical problem with a tube (*n* = 1). For 1 subject, the tube was located in the jejunum, and thus, ileal digestibility data will be presented for 8 subjects. Because the position of the tube does not impact dietary nitrogen utilization after intestinal absorption, other data were calculated for 9 subjects.

### Analyses

PEG-4000 concentration in the digesta was assessed by a turbidimetric method for the determination of the ileal flow rate [24]. The nitrogen content of digesta and the test meal was determined by an elemental analyzer (EA) using the Dumas method (Vario Micro Cube; Elementar) coupled to an isotopic ratio MS (IRMS; Isoprime, GV Instrument), enabling analysis of the ^15^N enrichment of digesta and the test meal. The elemental standard used was atropine (Thermo Electron), and the isotopic standard was L-glutamic acid (USGS41, Sigma-Aldrich).

Nitrogen digestibility and utilization were assessed using ^15^N recovery in digesta, plasma, and urine urea pools and in the test meal. Urea content in plasma and urine was measured by a colorimetric method on an external platform. The method adapted from Preston and McMillan [25] was used to isolate urea. For urines, sodium form of cation exchange resin (Dowex 50WX8 sodium form 100–200 mesh; Sigma-Aldrich) was used to isolate ammonia. Urea was then isolated from the supernatant on the resin after incubation with urease (Urease from Jack Bean type III; Sigma-Aldrich) at 30°C for 2 h. After acid precipitation with sulfosalicylic acid (10%), plasma urea fraction was treated with urease and separated from the AA fraction using the hydrogen form of cation exchange resin. The resins were eluted with KHSO_4_ (2.5 M), and the ^15^N enrichment of plasmatic and urinary urea in the supernatant was measured by EA-IRMS. The AA fraction, as well as the pellet obtained after acid precipitation containing plasma protein, were dried, and weighed before EA-IRMS measurement.

For AA quantification (other than tryptophan), 10-mg digesta or test meal was hydrolyzed with HCl 6N at 110°C for 24 h, and norvaline, added prior to hydrolysis, was used as an internal standard. To analyze sulfur AA in the test meal, performic acid oxidation was conducted before hydrolysis for the conversion of methionine and cysteine to their acid-stable derivatives, methionine sulfone and cysteic acid [26]. Tryptophan was analyzed in the test meal. Base hydrolysis (barium hydroxide 2N) was done for a 15 mg sample at 110°C for 24 h, and 5-methyl-tryptophan was used as an internal standard. Calibration standards were AA mixture (Waters) with the addition of specific AAs (norvaline, methionine sulfone, cysteic acid, tryptophan, 5-methyl-tryptophan). Hydrolysates and standards were derivatized following the manufacturer’s protocol with the AccQ-Tag Ultra Derivatization Kit (Waters). An Acquity Hclass ultra-high-performance liquid chromatography (U-HPLC) system with a photodiode array detector (PDA detector, Waters) was used to perform AA quantification. The AAs were separated with an AccQ-Tag AA C18 column (2.1 × 100 mm; 1.7 μm bead size; Waters) and quantified as mmol/g of dry matter. Plasma AA concentrations were assessed by U-HPLC on samples that were deproteinized by sulfosalicylic acid and using norvaline as an internal standard. After derivatization, the AA concentrations were analyzed with the AccQ-Tag Ultra Derivatization Kit (Waters).

To measure AA ^15^N enrichment in the ileal contents, 90-mg digesta and test meal were hydrolyzed with HCl 6N at 110°C for 24 h. AAs were isolated using a hydrogen form resin (Dowex 50WX8 hydrogen form 100–200 mesh; Sigma-Aldrich), and they were derivatized with ethyl chloroformate [27]. ^15^N enrichment of isolated AAs was analyzed by gas chromatography (GC 7890B; Agilent Technologies) coupled with an isotope ratio mass spectrometer (Vision, Elementar) via the GC5 interface (Elementar). The combustion furnace temperature was 950°C. The GC column (RXI-17, 30-m length, 0.25-μm inner diameter, 0.5-μm film thickness; Restek) temperature program started at 150°C, increased by 4°C per minute up to 200°C and by 25°C per minute up to 270°C, with the final temperature being maintained for 10 min. The inlet temperature was set at 270°C.

### Calculations

(1) *Ileal flow rate (F) (mL/30 min):*

$$F=\frac{{\left[\text{PEG}\right]}_{\text{solution}}}{{\left[\text{PEG}\right]}_{\text{digesta}}}\times \text{perfusion}\;\text{flow}\;\text{rate}\times 30$$

Here, [PEG] is the concentration of PEG in the infused solution and in the digestive contents, and the perfusion flow rate of PEG was set at 1 mL/min.

(2) *Total nitrogen flow rate (mmol/30 min):*

$$N_{\text{tot}\;\text{ileum}}=\frac{N_{s}\times {\text{DM}}_{s}\times F}{14\times 10}$$

Here, N_s_ is the percentage of nitrogen measured in the ileal sample, DM_s_ is the dry matter content of the ileal sample (g/100 mL), F is the ileal flow rate (mL/30 min), and 14 is the molar mass of nitrogen (g/mol).

(3) *Dietary nitrogen flow rate (mmol/30 min):*

$$N_{\text{diet}\;\text{ileum}}=N_{\text{tot}\;\text{ileum}}\times \frac{{\text{APE}}_{\text{ileum}}}{{\text{APE}}_{\text{meal}}}$$

Dietary nitrogen flow rate describes the amount of nitrogen from the ingested faba bean protein reaching the ileum. Here, N_tot_ _ileum_ is the total nitrogen flow (mmol/30 min), and APE (atom percent excess) the enrichment excess compared with basal enrichment (in AP) of ^15^N. Basal enrichment is the enrichment measured in the t = 0 sample from each volunteer.

(4) *True ileal digestibility of nitrogen (TID) (%N ingested):*

$$\text{TID}=\frac{N_{\text{ingested}}-\sum N_{\text{diet}\;\text{ileum}}}{N_{\text{ingested}}}\times 100$$

Here, N_ingested_ is the amount of nitrogen ingested by the subjects in the meal (mmol) and ΣN_diet ileum_ is the cumulative dietary nitrogen recovered in the ileum over the 8 h of the experiment (mmol).

(5) *Ileal dietary AA flow rate (mmol):*

$${\text{AA}}_{\text{diet}\;i}\left(t\right)={\left[\text{AA}\right]}_{\text{ileum}\;i}\left(t\right)\times \text{DM}\;\left(t\right)\times F\left(t\right)\times \frac{{\text{APE}}_{\text{ileum}\;i\;}\left(t\right)}{{\text{APE}}_{\text{protein}}}\text{,}$$

where [AA]_ileum i_(t) is the quantity of each AA “i” in the digestive contents at each period “t” (mmol/g), DM is the amount of dry matter in the digesta (g/100 mL), F is the ileal flow rate (mL/30 min), APE is the excess enrichment of each AA “i” in the digestive contents at each period “t” compared with the basal enrichment (in AP) of ^15^N and APE_protein_ that of faba bean protein. Basal enrichment is the enrichment measured in the t = 0 sample from each volunteer.

(6) *True ileal AA digestibility (%AA ingested):*

$${\text{TID}}_{AAi}=\frac{{\text{AAi}}_{\text{ingested}}-\sum {\text{AAi}}_{\text{diet}\;\text{ileum}}}{{\text{AAi}}_{\text{ingested}}}\times 100\text{,}$$

where AAi_ingested_ is the amount of AAi ingested by the volunteers in the meal (mmol), and ΣAAi_diet ileum_ is the cumulative dietary AAi recovered in the ileum over the 8 h of the experiment (mmol). Average AA digestibility was calculated from the mean of AA digestibilities weighted by the proportion of each AA in the protein. True ileal digestibility was assessed for 15 AAs and could not be calculated for tryptophan and cysteine because of their low recovery or absence in the digesta hydrolysates produced for gas chromatography-combustion IRMS.

(7) *DIAAS, the lowestDIAA ratio:*

$$\text{DIAA}\;\text{ratio}={\text{CS}}_{i}\times {\text{AA}\;\text{ileal}\;\text{digestibility}}_{i}\text{,}$$

where CSi is the chemical score of the AAi (i = 1–9)

$$\text{CS}=\frac{\text{mass}\;\text{of}\;{\text{IAA}}_{i}\;\text{in}\;1g\;\text{of}\;\text{dietary}\;\text{protein}\;}{\text{mass}\;\text{of}\;\text{the}\;\text{same}\;{\text{IAA}}_{i}\;\text{in}\;1g\;\text{of}\;\text{reference}\;\text{protein}}$$

The protein mass was calculated using both the specific N-to-protein conversion factor of 5.4 for legumes and the default conversion factor of 6.25 [28].

The reference AA profile used for the calculation was the requirement pattern of the older child, adolescent, and adult defined in the 2013 FAO report [6].

(8) *Dietary nitrogen transferred to body and urinary urea (mmol):*

$$N_{\text{diet}\;\text{urinary}\;\text{urea}}\left(t\right)={\text{Urine}\;\text{volume}\;\left(t\right)\;x\;\left[\;\text{urea}\right]\left(t\right)x\;}_{2}\times \frac{{\text{APE}}_{s}\left(t\right)}{{\text{APE}}_{\text{meal}}}$$

Here, [urea] is the concentration of urea in mmol/L, and the factor 2 the number of N in urea.

$$N_{\text{diet}\;\text{body}\;\text{urea}}\left(t\right)=\text{TBW}\;x\;\left[\text{urea}\right]\left(t\right)x\;2/0.92\times \frac{{\text{APE}}_{s}\left(t\right)}{{\text{APE}}_{\text{meal}}}$$

where TBW is the total body water in L, calculated by the equation of Watson et al. [29], [urea] is the plasma urea concentration in mmol/L, and 0.92 the concentration of water in the plasma.

(9) *NPPU (% of ingested N):*

$$\text{NPPU}=\frac{N_{\text{ingested}}-\left({\sum N}_{\text{diet}\;\text{ileum}}+{\sum N}_{\text{diet}\;\text{urinary}\;\text{urea}}+N_{\text{diet}\;\text{body}\;\text{urea}}\right)}{N_{\text{ingested}}}\times 100$$

NPPU is the amount of nitrogen retained in the body after 8 h. Here, N_diet body urea_ is the remaining dietary nitrogen in the body urea at 8 h.

#### Statistical analysis

The kinetics were analyzed with a mixed linear model with time as a repeated factor, followed by post hoc multiple comparisons with Bonferroni correction, to assess any difference from the baseline at each time point (SAS 9.4, SAS Institute).

## Results

### AA composition and ^15^N enrichment in faba bean

**FIGURE 2:**
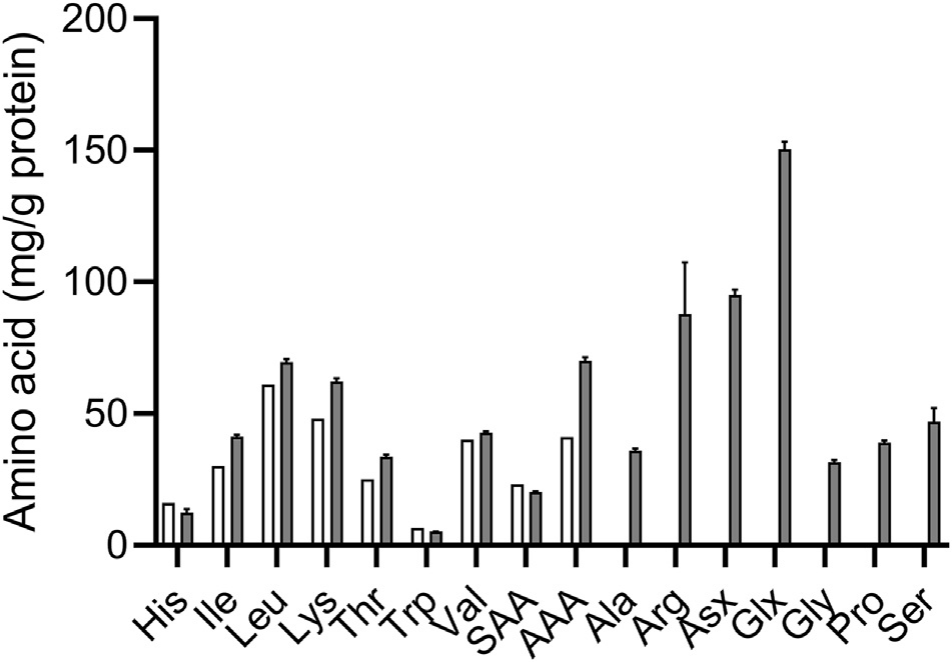
Amino acid composition of the faba bean meal (gray); for indispensable amino acids the reference protein pattern (children aged >3 y and adults) is shown (white) [30]. Values are means and SDs of 3 replicates. AAA, aromatic amino acids (Phe+Tyr); Asx, asparagine + aspartate; Glx, glutamine + glutamate; SAA, sulfur amino acids (Met+Cys). N × 6.25 was used to quantify protein amount.

Figure 2 shows the AA composition of the meal and the IAA with respect to the reference AA pattern (for children aged >3 y and adults) [30]. Histidine and tryptophan quantities were slightly below the reference pattern. The enrichment was homogeneous among AAs (data not shown), the mean value being 0.76 APE and the range from 0.64 APE for histidine to 0.86 APE for serine.

### Participants

Participants were, on average, of normal weight, aged 33 y, and mostly men (Table 1).

**TABLE 1:** Characteristics of the participants involved in the clinical study on ileal digestibility of faba bean

Characteristic	
Sex	7 males/2 females
Age (y)	33.2 ± 9.8
Height (cm)	174.2 ± 2.6
Weight (kg)	75.1 ± 9.5
BMI (kg/m^2^)	24.7 ± 2.6

Values are means ± SDs.

### Digestive kinetics and digestibility

**FIGURE 3:**
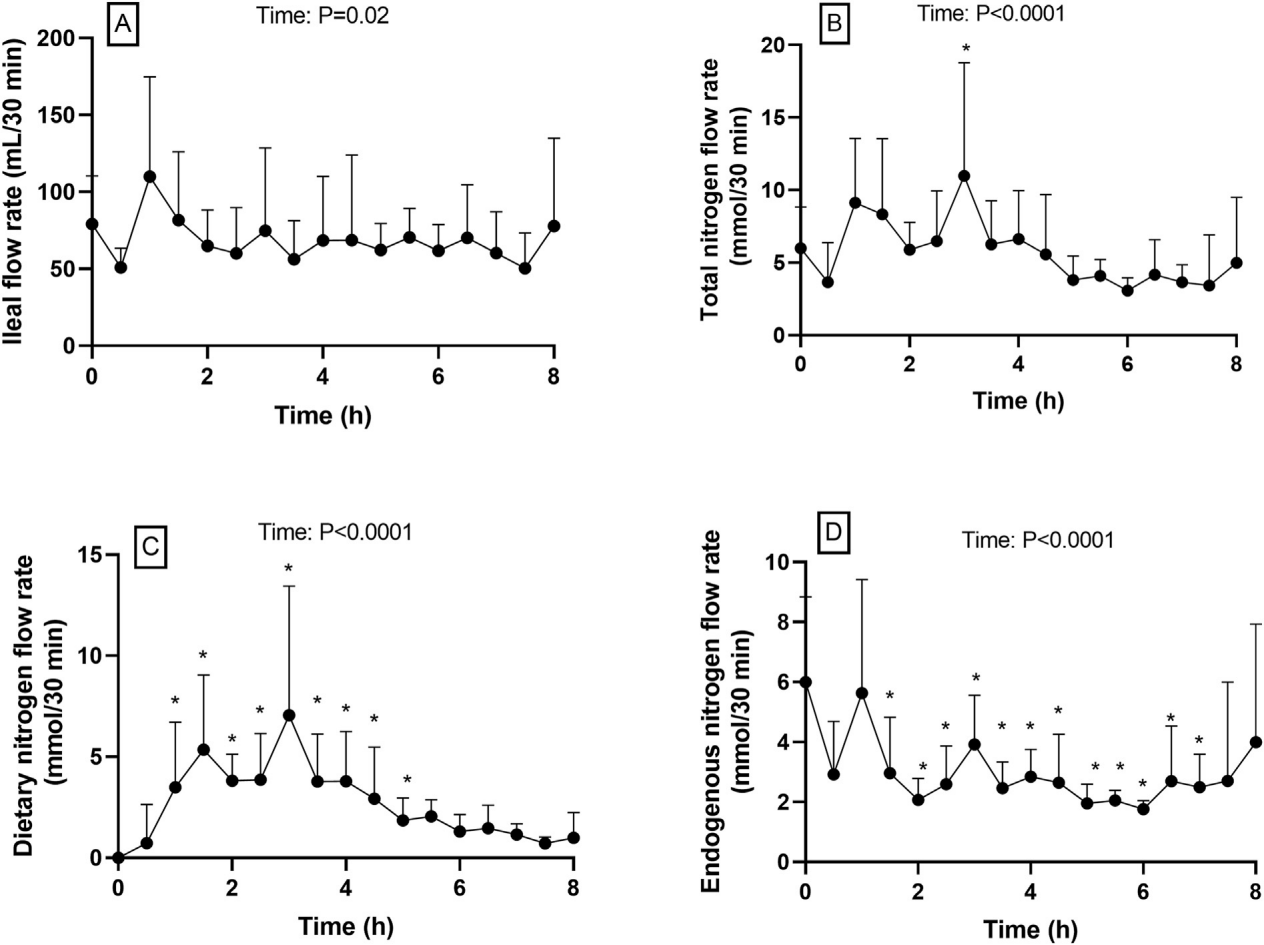
Flow rates of (A) digesta at the ileum site, including the intestinal perfusion of PEG-4000, (B) total nitrogen, (C) dietary nitrogen, and (D) endogenous nitrogen flow rates after ingestion of faba beans. Values are means ± SDs, *n* = 8. ∗Significant difference from baseline (mixed model with time as repeated factor, post hoc multiple comparisons with Bonferroni correction).

The mean ileal flow rate fluctuated through time (*P* = 0.02) from 79 ± 31 mL/30 min at baseline to 110 ± 65 mL/30 min after 1 h (Figure 3A**)**. In the subject with the tube located in the jejunum (data not shown), the flow rate reached a maximum of 479 mL/30 min at 30 min after meal ingestion and then fluctuated between 100 and 300 mL/30 min.

Total nitrogen flow was 6.0 ± 2.8 mmol/30 min at baseline and increased to a maximum of 11.0 ± 7.8 mmol/30 min at 3 h after meal intake (Figure 3B). The flow rate stabilized after 5 h. There was a significant effect of time and a significant difference from baseline at 3 h. Dietary nitrogen appeared in the terminal ileum on average 30 min after the meal was ingested. The maximum was observed between 1.5 h (5.4 ± 3.7 mmol/30 min) and 3 h (7.1 ± 6.4 mmol/30 min) (Figure 3C). After 5 h, the flow was not different from the baseline, being <2 mmol/30 min and decreasing to 1.0 ± 1.3 mmol/30 min at 8 h. At baseline, the endogenous nitrogen flow was 6.0 ± 2.8 mmol/30 min, and it significantly decreased to 2–4 mmol/30 min between 1.5 and 7 h (Figure 3D).

The cumulative amount of dietary nitrogen recovered at the terminal ileum 8 h after the meal was 43.0 ± 19.5 mmol from the 274 ± 15 mmol nitrogen ingested. Thus, the true ileal digestibility of protein was 84.1% ± 7.7%. In the subject with the tube in the jejunum, the digestibility was only 56% due to the high amount of unabsorbed nitrogen at this level of the intestine.

Digestibility varied from 85.1% ± 13.7% for histidine to 94.2% ± 3.6% for Glx (Table 2). The mean digestibility of individual AAs was ∼6 percentage points higher than the digestibility of nitrogen, reaching 89.8% ± 5.9%, whereas the mean IAA digestibility was 88.0% ± 7.3%.

**TABLE 2:** True ileal amino acid and nitrogen digestibility of faba bean protein (%)

Amino acid	Digestibility (%)
Mean IAA (weighted)	88.0 ± 7.3
Histidine	85.1 ± 13.7
Isoleucine	88.4 ± 7.2
Leucine	87.6 ± 7.1
Valine	88.4 ± 6.7
Lysine	91.6 ± 5.4
Methionine	92.4 ± 5.8
Phenylalanine	90.1 ± 7.1
Threonine	89.2 ± 6.5
Mean DAA (weighted)	90.3 ± 5.5
Alanine	89.3 ± 6.3
Glycine	86.9 ± 8.4
Asx	89.2 ± 6.4
Glx	94.2 ± 3.6
Proline	87.7 ± 7.0
Serine	89.3 ± 6.1
Tyrosine	86.7 ± 8.9
Mean all AA (weighted)	89.8 ± 5.9
Nitrogen	84.1 ± 7.7

Data are shown as means ± SDs, *n* = 8.

Abbreviations: IAA, indispensable amino acid; DAA, dispensable amino acid; Asx, asparagine + aspartate; Glx, glutamine + glutamate.

#### Chemical score, PDCAAS, and DIAAS

Table 3 shows quality indices of faba bean, calculated taking into account a N-to-protein conversion factor of 5.4 (specific for legumes) or 6.25 (used as default) [28]. Regarding the chemical score and PDCAAS, histidine and tryptophan were deficient. PDCAAS and DIAAS values were rather similar. Assuming that tryptophan digestibility was similar to that of other AAs, histidine and tryptophan were deficient at the same level, resulting in a score ranging between 0.66 and 0.67 for a N-to-protein conversion factor of 6.25 and 0.76–0.77 for a factor of 5.4.

**TABLE 3:** Quality scores of faba bean protein

	Chemical score		PDCAAS		DIAAS	
	N × 5.4	N × 6.25	N × 5.4	N × 6.25	N × 5.4	N × 6.25
Histidine	0.90	0.78	0.76	0.66	0.77	0.67
Isoleucine	1.59	1.37	1.34	1.16	1.41	1.22
Leucine	1.32	1.14	1.11	0.96	1.16	1.00
Lysine	1.50	1.30	1.26	1.09	1.38	1.19
Sulfur amino acids	1.02	0.88	0.86	0.74	0.94	0.81
Aromatic amino acids	1.98	1.71	1.66	1.44	1.78	1.54
Threonine	1.56	1.34	1.31	1.13	1.39	1.20
Tryptophan	0.91	0.78	0.76	0.66	—	—
Valine	1.23	1.07	1.04	0.90	1.09	0.95
Score	0.90	0.78	0.76	0.66	0.77	0.67

Values are based on digestibility data of 8 subjects.

Abbreviations: DIAAS, digestible indispensable amino acid score; PDCAAS, protein digestibility corrected amino acid score.

### Metabolic kinetics and losses

**FIGURE 4:**
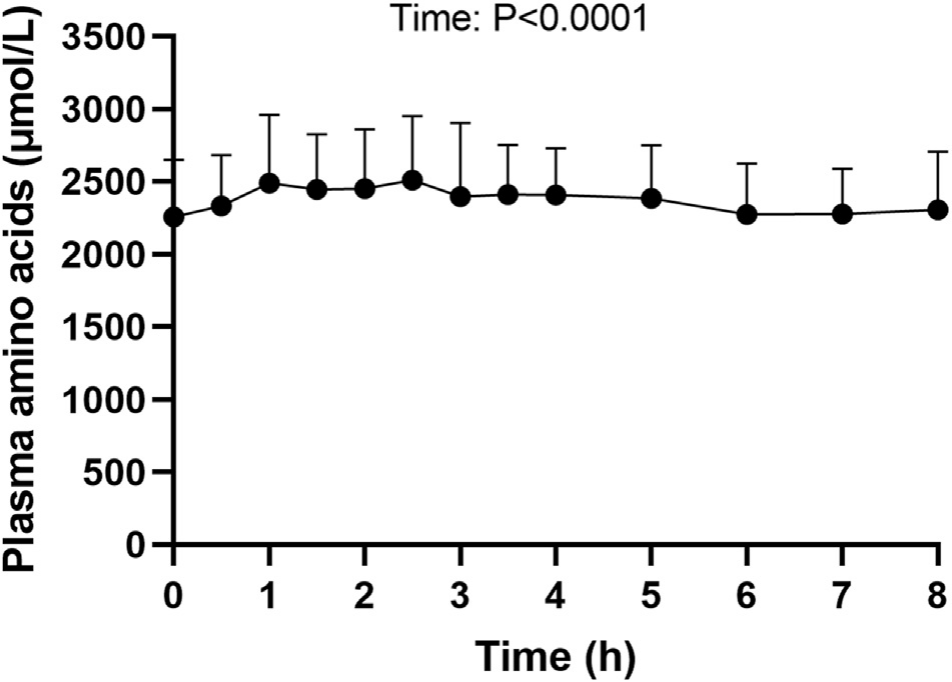
Plasma total amino acid concentration after ingestion of faba beans. Values are means ± SDs, *n* = 9. The effect of time was analyzed in a mixed model with time as repeated factor.

The plasma AA concentration (Figure 4) moderately fluctuated from 2256 ± 393 μmol/L at baseline to 2500 μmol/L between 1 and 2.5 h, with a significant effect of time (*P* < 0.0001) but no difference between baseline and each time point after Bonferroni correction for multiple comparisons.

**FIGURE 5:**
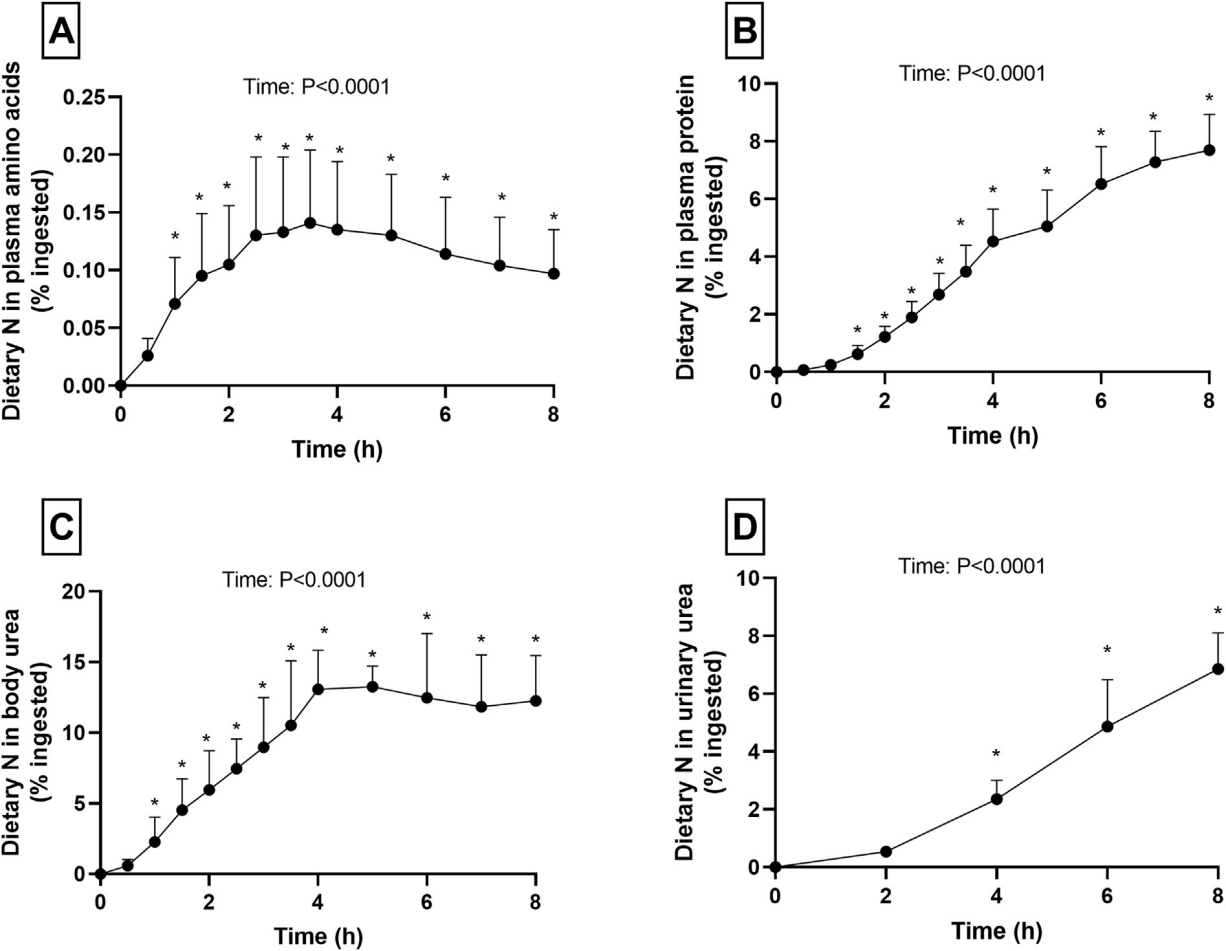
Transfer of dietary nitrogen in plasma nitrogen pools and urinary urea after ingestion of faba beans. (A) Plasma amino acids. (B) Plasma protein. (C) Plasma urea. (D) Urinary urea. Values are means ± SDs, expressed as proportion (%) of ingested nitrogen, *n* = 9. ∗Significant difference from baseline (mixed model with time as repeated factor, post hoc multiple comparisons with Bonferroni correction).

The appearance of dietary nitrogen in plasma AAs (Figure 5A) increased during the first 3.5 h and then slightly decreased but was still significantly higher from the baseline at 8 h (*P* < 0.0001). The incorporation of dietary nitrogen in the plasma protein pool (Figure 5B) was still increasing at 8 h and represented 7.7% ± 1.2% of ingested nitrogen. The transfer to body urea increased for 4 h and then was stable. At 8 h, 12.3% ± 3.2% of nitrogen was still present in body urea (Figure 5C). The cumulated loss of dietary nitrogen in urine during the 8 postprandial hours accounted for 5.7% ± 1.0% of ingested nitrogen **(**Figure 5D**).** For all these parameters, there was a significant effect of time (*P* < 0.0001).

The deamination losses are the sum of ^15^N lost in urinary urea (18.7 ± 3.6 mmol) and that still present in body urea after 8 h (33.6 ± 9.4 mmol), representing 19.2% ± 3.6% of ingested nitrogen. The NPPU was calculated from the sum of digestive (43.0 ± 20.6 mmol) and deamination losses (52.3 ± 10.6 mmol), being 64.7% ± 9.7% of ingested nitrogen.

## Discussion

We aimed to evaluate the protein quality of cooked, dehulled faba beans in healthy humans using the nasoileal intubation method combined with ^15^N intrinsically labeled protein. The method allows separation of dietary (exogenous) and endogenous AA losses and determination of the true ileal digestibility of AAs. We observed that digestibility of faba bean protein was moderate (∼85%), but mean AA digestibility was good (∼90%). Overall protein quality was restricted by the limited histidine and tryptophan content.

Compared with other protein sources assessed in similar conditions using ^15^N labeling and ileal tubes, the obtained nitrogen digestibility of faba bean was moderate (84%). The highest observed nitrogen digestibility in such studies has been for total milk protein and casein (95%) [15,16,31] and the lowest for raw egg white (50%) [32]. The legume proteins previously tested using this method have been in the form of flours, concentrates, or isolates and showed nitrogen digestibility of 89% for pea flour [10], 91% for lupin flour [12], 92% for pea protein isolate [13], 92% for soy isolate [12], and 94% for pea globulin [11]. However, using the ileal balanced approach in humans, no data exist on legumes ingested in forms other than those mentioned above. In pigs, the standardized ileal nitrogen digestibility of intact mung beans and adzuki beans was ∼80% [33], but lower values have been obtained for whole faba beans, varying from 70% to 80%, depending primarily on the tannin content of the cultivar [34].

The digestibility of individual faba bean AAs was homogeneous, that is, close to the mean AA digestibility (89.8%), ranging from 85% for histidine to 94% for glutamine/glutamate (Glx). Interindividual variation was high, especially for histidine. Ileal AA digestibility was ∼6 percentage points higher than nitrogen digestibility. In pigs, lower values were reported for ileal AA digestibility of faba bean, with the lowest digestibility found for methionine (from 58% to 77%, depending on the cultivar) and glycine (from 59% to 73%) [34]. In humans, digestibility values of individual AAs for legumes consumed as seeds have been obtained with the double tracer method [9], for which no internal validation exists [13]. Regarding Moroccan dehulled faba beans cooked in a soup, a high variation in the digestibility values of individual AAs was present; the lowest AA digestibility was observed for threonine (44%) and the highest for valine (69%) [35]. In the current study, the digestibility of individual IAAs was within 7 percentage points, ranging from 85% to 92%. Overall, in the Moroccan study [35], the mean faba bean IAA digestibility was 61.1% ± 5.2%, which is much lower than in our study (88.0% ± 7.3%). The discrepancies between these results may be due to the different cultivars, as discussed later, and to the different methods used. It has been speculated that the dual isotope method underestimates the IAA digestibility compared with the ileal balance method [35,36], but another internal comparison study does not support this systematic underestimation [37] as sometimes the dual isotope method seems to overestimate and sometimes underestimate digestibility compared with the direct method. Regarding legumes, no internal comparison exists; thus, external comparisons include the effect of the model or method and the effect of the cultivar. For instance, in the study of Thomas et al. [38], the IAA digestibility of whole Jamaican red kidney beans obtained using the dual isotope method was 79.4% ± 0.5%, being 6 percentage points higher than reported in pigs [39]. For other legumes consumed as cooked seeds, Kashyap et al. [9] showed that the digestibility values for whole chickpea and yellow pea measured by a dual isotope method were below (71%–75%) those reported by Han et al. [39] in pigs, that is, 79% for mung bean and 83% for chickpea.

Generally, plant-based proteins have lower nutritional quality (DIAAS < 0.75) than animal-based proteins [40]. Based on the IAA composition of faba bean, the calculated chemical score showed that histidine and tryptophan were marginally deficient, using both legume-specific (5.4) and general N-to-protein conversion factors (6.25) [28]. DIAAS was 0.77 with a factor of 5.4 and 0.67 using the default factor of 6.25. Histidine and tryptophan were the first limiting AAs, but sulfur AAs also had a DIAA ratio of <1, whereas the sulfur AA chemical score was slightly >1. The DIAA ratio, as such, could not be analyzed for tryptophan, but it was approximated using the mean digestibility of the other AAs. Interestingly, the PDCAAS was very similar to the DIAAS due to the low variation of digestibility values among AAs. It is noteworthy that in the Moroccan study [35], faba bean digestibility values could not be determined for histidine, tryptophan, and methionine, which were critical AAs in our study. A previous study using the ileal balance method [13] showed that DIAAS values for pea protein isolate, unsurprisingly, were better than those of legumes consumed as seeds. The DIAAS was 1.00 when the conversion factor of 6.25 was used and 1.16 when the legume-specific factor of 5.4 was used [13].

The dietary nitrogen transfer to body urea (12.3%) was similar to values observed in other studies (pea protein 10.7%, casein 14.7%) and to urinary urea slightly lower (5.7%) than previously observed [13,41]. Concerning the postprandial handling of nitrogen and AAs after absorption, metabolic losses for faba bean were in the low range (19%). In other studies measuring the NPPU after a bolus, the deamination losses were the lowest for total milk protein (10%–15% depending on whether fat or sucrose was co-ingested) [15], lupin flour (17%) [12], soy protein (18%) [12], and whole eggs (18%) [42], whereas the highest values were observed for gluten (25%) [16] and whey protein (28%) [41]. Postprandial deamination is mainly influenced by digestion kinetics, the presence of other nutrients in the meal, and the AA profile of the protein. For example, the unbalanced AA profile in gluten, where lysine is markedly limiting, led to increased deamination [16], whereas for whey protein, deamination was a consequence of fast digestion kinetics [41]. The presence of carbohydrates in the meal has been shown to spare nitrogen [15,43] in association with insulin release, which activates protein synthesis pathways. As the AA profile of faba bean proteins in our study was marginally unbalanced and the protein was consumed in the original matrix (mashed dehulled bean) containing fiber and carbohydrates, this resulted in an intermediate slow digestion. This is an important issue related to the potential benefits of consuming beans as seeds rather than as protein isolates. Due to digestive losses, the NPPU of faba bean (65%) was similar to that of gluten (66%) [16] but lower than of those of casein (71%) [13], pea protein (72%–79%) [10,11,13], or milk protein (80%–85%) [15].

Faba bean is a widely consumed food in Africa, Asia, and Mediterranean regions but much less so in Western countries [19]. It is noteworthy that the nutritional values of the cultivars adapted in various regions can differ. Jezierny et al. [34] showed that in pigs, threonine digestibility ranged 13 percentage points and lysine 8 percentage points among the 6 faba bean cultivars tested, with high tannin content being the main determinant of low digestibility. The Moroccan faba bean soup tested in humans was deficient in sulfur AAs and threonine but was rich in lysine [35]. This is advantageous in cereal-rich diets in developing countries, where lysine is usually deficient. Tannins, phytic acid, and trypsin inhibitors have a negative effect on legume protein digestibility [19,44,45], and different processing methods, such as those used in our bean mash processing, decreases their contents. Dehulling removes tannins, phytic acid starts to degrade during soaking, and cooking (thermal treatment) inactivates trypsin inhibitors [19]. Thus, these factors, in addition to the potential differences between the cultivars, should be considered when assessing faba bean protein digestibility.

The main strength of this study is that the protein and AA digestibility were evaluated in dehulled beans, as widely consumed, using the direct method with ^15^N labeled protein and nasoileal tubes. In addition to the ileal digestibility of AAs, the ^15^N labeling allowed us to obtain a complete picture of digestive and metabolic bioavailability of dietary nitrogen. Although the ^15^N balance method is a standard method for determining protein and AA bioavailability, it has several limitations that were detailed in a recent review [7]. In particular, the invasiveness of the tube method leads to a high dropout rate and makes it difficult to collect a complete dataset for a food product. Moreover, a slight overestimation of digestibility can be expected due to incomplete digestion after 8 h (although marginal at this time), and on the contrary, a 1.5% underestimation of digestibility can result from the recycling of the tracer in the intestinal lumen [46]. Also, urea recycling in the hindgut has been shown to contribute to ∼35% of urea production, which depends on the nutritional status and is especially activated when protein intake is not sufficient [[47], [48], [49]]. Using this putative contribution of 35%, the residual ^15^N in body urea at 8 h (12% of ingested) and subsequently the deamination could thus be overestimated by ∼4%, a value that warrants further investigation.

To conclude, this study determined the nitrogen and AA bioavailability of faba bean ingested as cooked, dehulled mash using the ^15^N ileal balance method. Faba bean nitrogen digestibility was moderate, but AA digestibility was close to 90%. Postprandial nitrogen losses through deamination were limited. The overall protein quality was restricted by digestive losses and by the limited histidine and tryptophan contents.

## Acknowledgments

We thank Bruna Neves from the Human Nutrition Research Center of Avicenne Hospital for her contribution to the clinical trial and Dr. Antti Knaapila from the University of Helsinki for his contribution to the processing and sensorial evaluation of the beans. Jaakko Haarala, Aino Hämäläinen, and Tuuli Markkanen are thanked for their technical help with the ^15^N fertilization and harvesting of faba beans. We thank Outi Brinck from the University of Helsinki for technical support regarding the processing of the beans.

## Author contributions

The authors’ responsibilities were as follows – A-MP acquired funding; STI, JC, RB, A-MP, CG designed the research; FS and AS designed and supervised the ^15^N labeling of faba beans; STI, JC, CG, MC, GA, NK, MS conducted the research; STI, JC, CG analyzed the data; STI wrote the manuscript draft; STI and CG had primary responsibility for the final content; STI, CG, JC, AS, FS, and A-MP reviewed and edited the manuscript; and all authors: read and approved the final manuscript.

### Conflicts of interest

The authors have no conflicts of interest to declare. The funders had no role in study design or analysis or in the writing of this article.

### Funding

The Leg4Life (Legumes for Sustainable Food System and Healthy Life) project was funded by the Strategic Research Council at the Research Council of Finland (Grant numbers 327698 and 352481).

### Data sharing

Data described in the manuscript, code book, and analytic code will be made available upon request, pending application and approval.

## Appendix A Supplementary data

Multimedia component1 Multimedia component1
