# Supplementary material for: True Ileal Amino Acid Digestibility and Protein Quality of 15N-Labeled Faba Bean in Healthy Humans
Source: J Nutr. 2024 Feb 3;154(4):1165–74. doi: 10.1016/j.tjnut.2024.01.030 (PMC11007748; doi:10.1016/j.tjnut.2024.01.030)
Supplement: Multimedia component1 [file mmc1.pdf]

Itkonen ST et al. True ileal amino acid digestibility and protein quality of $^{15}N$-labeled faba bean in healthy humans.

Supplementary Material.

Supplemental Table. Nitrogen percent (%N) and $^{15}N$ enrichment in atom percent in whole, dehulled and soaked beans and in bean mash (n=3).

| | %N [mean (sd)] | Protein (g/100 g dry matter) | $^{15}N$ atom percent enrichment |
|---|---|---|---|
| Whole beans | 5.2 (0.31) | 27.9 | 1.23 |
| Dehulled beans | 5.6 (0.04) | 30.3 | 1.36 |
| Soaked beans | 5.7 (0.15) | 30.7 | 1.25 |
| Bean mash | 5.7 (0.05) | 30.7 | 1.27 |
